# Long noncoding RNA FER1L4 promotes the malignant processes of papillary thyroid cancer by targeting the miR-612/ Cadherin 4 axis

**DOI:** 10.1186/s12935-021-02097-2

**Published:** 2021-07-21

**Authors:** Luyao Wu, Yu Ding, Houchao Tong, Xi Zhuang, Jingsheng Cai, Yan Si, Hao Zhang, Xiaoting Wang, Meiping Shen

**Affiliations:** 1 Department of General Surgery, the First Affiliated Hospital of Nanjing Medical University, 300 Guangzhou Road, Nanjing, 210029 Jiangsu China; 2 Department of Thyroid and Breast Surgery, Changzhou No. 2 People’s Hospital Affiliated to Nanjing Medical University, Changzhou, China; 3 Department of General Surgery, Yixing People’s Hospital, Yixing, China

**Keywords:** Papillary thyroid cancer, Long noncoding RNA, FER1L4, MiR-612, Cadherin 4

## Abstract

**Background:**

Long noncoding RNAs (lncRNAs) have emerged as crucial regulators in various cancers. However, the functional roles of most lncRNA in papillary thyroid cancer (PTC) are not detailly understood. This study aims to investigate the biological function and molecular mechanism of lncRNA Fer-1 like family member 4 (FER1L4) in PTC.

**Methods:**

The expression of FER1L4 in PTC was determined via operating quantitative real-time PCR assays. Meanwhile, the clinical significance of FER1L4 in patients with PTC was described. The biological functions of FER1L4 on PTC cells were evaluated by gain and loss of function experiments. Moreover, animal experiments were performed to reveal the effect on tumor growth. Subcellular distribution of FER1L4 was determined by fluorescence in situ hybridization and subcellular localization assays. Luciferase reporter assay and RNA immunoprecipitation assay were applied to define the relationship between FER1L4, miR-612, and Cadherin 4 (CDH4).

**Results:**

Upregulated expression of FER1L4 in PTC tissues was positively correlated with lymph node metastasis (*P* = 0.020), extrathyroidal extension (*P* = 0.013) and advanced TNM stages (*P* = 0.013). In addition, knockdown of FER1L4 suppressed PTC cell proliferation, migration, and invasion, whereas ectopic expression of FER1L4 inversely promoted these processes. Mechanistically, FER1L4 could competitively bind with miR-612 to prevent the degradation of its target gene CDH4. This condition was further confirmed in the rescue assays.

**Conclusions:**

This study first demonstrates FER1L4 plays an oncogenic role in PTC via a FER1L4-miR-612-CDH4 axis and may provide new therapeutic and diagnostic targets for PTC.

**Supplementary Information:**

The online version contains supplementary material available at 10.1186/s12935-021-02097-2.

## Background

Thyroid cancer (TC) is the most frequent malignancy of the endocrine system and ranks ninth in the incidence of tumors worldwide [1]. Papillary thyroid carcinoma (PTC) is the major pathological type, accounting for more than 85% of differentiated thyroid cancer (DTC) [2]. With the improvements of diagnostic technologies such as ultrasound, the detection rate of DTC has been significantly increased during the last few decades [3]. However, patients with DTC usually have an excellent prognosis, with a 10-year disease-specific survival rate of over 90% [4]. Of note, about 10% of patients with DTC have distant metastases to lungs or bones at diagnosis or during follow-up, resulting in a poor prognosis [5]. Meanwhile, the incidence of larger tumors also elevates, which is not likely to be explained by the screening effect [3]. And to date, the etiology of thyroid cancer remains unclear. Thus, exploring the molecular basis of PTC pathogenesis and progression is crucial for developing more effective therapeutic and diagnostic targets for PTC.

Long noncoding RNAs refer to the noncoding portions of the genome without a protein-coding signature that transcript longer than 200 nucleotides [6]. LncRNAs are considered to regulate varieties of physiological and pathological processes, including the occurrence and progression of cancers [7, 8]. In recent years, aberrant expression of lncRNA has been found in multiple tumors. For example, in hepatocellular carcinoma, lncRNA-AY could promote hepatocellular carcinoma metastasis via induction of chromatin modification for ITGAV transcription [9]. In addition, it was reported that upregulated expression of lncRNA BCRT1 in breast cancer remarkably accelerated tumor growth and metastasis by acting as a sponge for miR-1303 [10]. However, researches on the underlying role of lncRNA in PTC carcinogenesis are still lacking yet.

LncRNAs perform their complex biological functions in multiple ways, which are closely related to their subcellular localization [11]. For example, lncRNA largely localized in the cytoplasm mainly functions by acting as a decoy for miRNA, in which lncRNA could control miRNA availability for its target gene, so named as competing endogenous RNAs (ceRNAs) [12]. In the case of thyroid cancer, lncRNA-GAS8-AS1, localized in the cytoplasm of PTC cells, was found that could promote the autophagy of PTC cells by sponging oncogenic miR-187-3p and miR-1343-3p, and therefore respectively upregulates the expression of ATG5 and ATG7 [13]. In this study, lncRNA Fer-1 like family member 4 (FER1L4) was identified as an oncogene that promoted the proliferation, migration, and invasion of PTC cells via functioning as ceRNA for tumor suppressor miR-612.

Cadherin 4, also named retinal cadherin, is a classical cadherin from the cadherin superfamily[14]. Cadherins, consisted of epithelial-cadherin (CDH1), neural-cadherin (CDH2), placental-cadherin (CDH3), and so on, are transmembrane glycoproteins responsible for cell–cell adhesion, tissue patterning, and carcinogenesis [15]. Previous researches indicate that cadherins could bind to catenin proteins, and then form the cadherin-catenin complex to activate the catenin signaling, as well as involve in the epithelial-mesenchymal transition (EMT) process [16]. However, the function of CDH4 in PTC remains unknown, and its role in the above processes is still controversial [17]. In the present study, CDH4 was considered as the downstream target of miR-612, which mediated the promoting role of FER1L4 in PTC cells.

## Methods

### Patients and tissue samples

Eighty papillary thyroid cancer tissues and adjacent normal tissues were collected from 80 patients with PTC who received operation from the First Affiliated Hospital of Nanjing Medical University (NMU). None of the patients underwent any other treatment but surgery. All collected tissue samples were immediately snap-frozen in liquid nitrogen and stored at − 80 °C until required. Our study was approved by the Ethics Committee of the First Affiliated Hospital of NMU.

### Cell lines

Four PTC cell lines (K-1, TPC-1, B-CPAP, and IHH-4) and a normal thyroid follicular epithelium cell line (Nthy-ori3-1) were purchased from the American Type Culture Collection (ATCC, Virginia, USA). K-1, B-CPAP, and Nthy-ori3-1 cell were cultured in RPMI1640 medium (Gibco, Carlsbad, CA, USA), while TPC-1 cell was cultured in DMEM with high glucose (Gibco, Carlsbad, CA, USA). A mixture (1:1) of RPMI1640 and DMEM was used to culture the IHH-4 cell line. 1% antibiotics (100 U/ml penicillin and 100 mg/ml streptomycin) and 10% fetal bovine serum (Gibco, Carlsbad, CA, USA) were added to all of mentioned culture media. All cell lines were incubated in a humidified atmosphere at 37 °C containing 5% CO2. All cell lines have been authenticated by short tandem repeat analysis, as well as tested for mycoplasma contamination before conducting this study.

### RNA extraction and quantitative real-time PCR analysis

Total RNA was extracted from tissues and cultured cell lines using TRIzol reagent (Invitrogen, MA, USA). A PrimeScript RT reagent kit (Takara, Kyoto, Japan) was used to synthesize cDNA. MiRNAs were reverse transcribed after polyadenylation using Revert Aid First Strand cDNA Synthesis Kit (Thermo Scientific, MA, USA). qRT-PCR was performed with AceQ qPCR SYBR Green Master Mix (Vazyme, Nanjing, China). Results were calculated using the 2^−ΔΔCT^ method and normalized to the expression of GAPDH for mRNA or U6 for miRNA. Primers used in the study were listed in Supplementary Table 1 (Additional file 1: Table S1).

### Cell transfection

PTC cells were transfected with 50 nm small interfering RNAs (siRNAs) and plasmid vectors when they grew to 30–40% density using Lipofectamine 3000 (Invitrogen, Carlsbad, CA, USA). In this study, siRNAs, miR-612 mimics, miR612 inhibitor, and correspondent negative control were purchased from Genepharma. After 48 h post-transfection, the cells were harvested for performing the following experiments. Plasmid vectors encoding FER1L4 and biologically active short hairpin RNAs (shRNA) targeting FER1L4 or CDH4 were generated either (Genepharma, Shanghai, China). Stable cell lines were obtained by using 2 μg/ml puromycin (Sigma-Aldrich, St-Louis, Missouri, USA) for about three weeks. Nucleotide sequences mentioned above were listed in Supplementary Table 2 (Additional file 1: Table S2).

### Cell proliferation assay

To investigate the effect on cellular proliferation of corresponding treatment, cell counting kit 8 (CCK8) assay, colony formation assay, and 5-Ethynyl-2′-deoxyuridine (EdU) incorporation assay was applied. Detailed information has been described before [18].

### Animal experiment

Four weeks old female BALB/c nude mice were purchased from the Animal Center of NMU, and all experiments were approved by the Committee on the Ethics of Animal Experiments of the Nanjing Medical University. For the tumorigenicity studies, a total of 20 mice were randomly assigned, and stably transfected cells (1 × 10^6^ cells/100 μl of phosphate buffer saline) were subcutaneously injected into the flank of nude mice. The tumor volume was measured every week and calculated by the formula: volume = (length × width^2^)/2.

### Immunohistochemical (IHC) analysis

All specimens were fixed in 4% formalin and then embedded in paraffin. After blocking endogenic peroxides and proteins, these sections were incubated with primary antibodies specific for Ki-67 (Abcam, Cambridge, MA, USA) or CDH4 (Abclonal, Wuhan, China) at 4 °C overnight, lastly counterstained with hematoxylin after incubating with the secondary antibodies at 37 °C for 1 h. Random images were obtained using a light microscope (Olympus Corp, Tokyo, Japan).

### Cell migration and invasion assays

Transwell chambers (Corning, New York, NY, USA) coated with or without Matrigel (BD Bioscience, Franklin Lakes, NJ, USA) were used to evaluate the function of genes on cellular migration and invasion. Meanwhile, cell motility was also examined by wound healing assay. Detailed information has been described before [18].

### Flow cytometric analysis

Treated cells were collected for flow cytometric analysis. According to the protocol (MultiSciences, Hangzhou, China), APC-Annexin V and Propidium Iodide (PI) were used to stain cells, and the rate of apoptosis was analyzed by a flow cytometer (FACScan, BD Biosciences, USA). For cell cycle analysis, treated cells were stained by PI-staining solution, and then the percentages of cells in the G_0_–G_1_, S, and G_2_–M phase were counted.

### Western blot assay and antibodies

Western blot assay was performed following the previous protocol. The primary antibody used were listed in Supplementary Table 3 (Additional file 1: Table S3).

### Subcellular fractionation and Fluorescence in situ hybridization (FISH)

The separation and purification of cytoplasmic and nuclear RNA were implemented using the PARIS Kit (Life Technologies, USA) according to the manufacturer’s instructions. For the FISH assay, the Cy3-labeled FER1L4 probes used in our study were synthesized (RiBo Ltd, Guangzhou, China). Briefly, the prepared cells were incubated with specific probes at 37 °C overnight after fixation and permeabilization. Finally, the nuclei were stained by DAPI and observed using a confocal laser scanning microscope (Zeiss LSM5 Live, Oberkochen, German).

### Dual-luciferase reporter assay

The sequences of FER1L4 and CDH4 3′-UTR containing wild-type or mutated miR-612 binding sites were synthesized and loaded into a pGL3 luciferase reporter vector (Promega, Wisconsin, USA). Then, TPC-1 cells (4 × 10^5^) were co-transfected with miRNA mimics and luciferase reporter vectors. After 48 h of incubation, the luciferase activities were measured using a Dual-Luciferase Reporter Assay System (Promega, Wisconsin, USA). Relative luciferase activity was normalized to *Renilla* luciferase.

### RNA immunoprecipitation (RIP) assay

To detect whether FER1L4 influences miR-612-dependent RNA-induced silencing complex, RIP assays were conducted using the Magna RIPTM RNA-binding protein immunoprecipitation kit (Millipore, USA). Briefly, the prepared cells were lysed and incubated with anti-Ago2 (Abcam, Cambridge, MA, USA) or IgG antibody at 4 °C overnight. Then, cell lysates were incubated with the protein A magnetic beads for 4 h. Finally, the coprecipitated RNAs were collected for qRT-PCR analysis.

### RNA pull-down assay

First, the biotinylated FER1L4 probe was synthesized (RiboBio, Guangzhou, China), and then the probe was mixed with C-1 magnetic beads (Life Technologies, Waltham, MA, USA) for 20 min at room temperature. According to the protocol, subsequently, collected magnetic beads were resuspended with cell lysate by slowly rotated at 4 °C for 2 h. Finally, the RNA complex bound to the beads was extracted and subjected to qRT-PCR analysis for relevant specific miRNA.

### Bioinformatic analyses

Gene expression data of PTC was extracted from the TCGA database and used to explore abnormally expressed lncRNA. MiRNAs targeted by FER1L4 were predicted using starbase (http://starbase.sysu.edu.cn/), miRcode (http://www.mircode.org/), and RegRNA2.0 (http://regrna2.mbc.nctu.edu.tw/). Meanwhile, miRWALK (http://zmf.umm.uni-heidelberg.de/apps/zmf/mirwalk/) was applied to determine the downstream targets of miR-612.

### Statistics analysis

The data were described by the mean ± standard deviation (SD) in three independents experiments. For comparing statistical differences, Student’s t-tests, Pearson Chi-square test, Wilcoxon test were performed as appropriate using SPSS v22.0 and GraphPad Prism 6. Spearman’s correlation analysis was selected to analyze the correlations among FER1L4, miR-612, and CDH4. A *P*-value less than 0.05 was considered statistically significant.

## Results

### FER1L4 was upregulated and associated with later-stage diseases in PTC

**Fig. 1:**
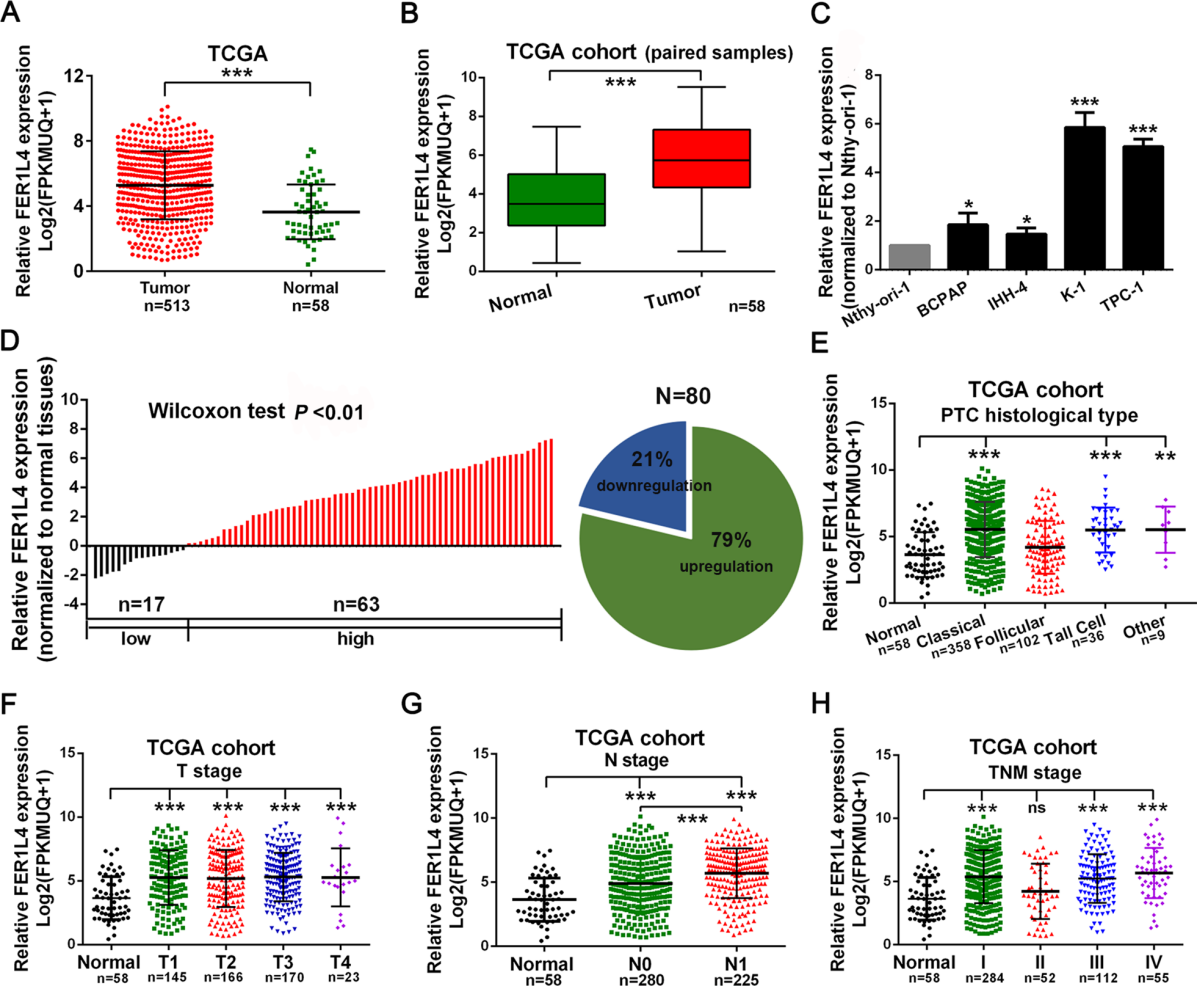
FER1L4 was upregulated in PTC and associated with later-stage diseases. **A**, **B** The expression of FER1L4 was upregulated in unpaired or paired PTC tissue samples based on the TCGA database. **C** An enhanced expression level of FER1L4 was detected in PTC cell lines. **D** qRT-PCR analysis of 80 paired PTC tissue samples. **E–H** Expression patterns of FER1L4 based on histological type, T stage, N stage, and TNM stage from TCGA database. Error bars, mean ± SD. NS, not significant. ** *P* < 0.01, *** *P* < 0.001

To identify potential drivers in PTC tumorigenesis, expression signatures of genes were screened based on the TCGA database. In this study, we observed preferential upregulation of lncRNA FER1L4 in thyroid cancer tissues (n = 513), compared with normal thyroid tissues (n = 58; Fig. 1A, B). Moreover, FER1L4 also showed concordance in a pattern of expression in PTC cell lines when compared with an immortalized thyroid follicular epithelium cell line Nthy-ori-1 (Fig. 1C). Meanwhile, an identical result was detected via measuring the expression of FER1L4 in 80 paired PTC tissues and adjacent normal tissues, in which 79 percent of patients exhibited higher expression of FER1L4 (Fig. 1D). Furthermore, through investigating the clinical significances of FER1L4 in these 80 PTC patients, it showed enhanced expression of FER1L4 was correlated with higher lymph node metastasis rate (*P* = 0.020), higher potential of extrathyroidal extension (*P* = 0.013), and advanced TNM stages (*P* = 0.013) (Table 1). In addition, analysis of the TCGA cohort suggested that upregulation of FER1L4 also predicted higher lymph node metastasis rate (N stage) in patients with PTC (Fig. 1E-H). Herein, these results indicated upregulation of FER1L4 was associated with later-stage diseases in PTC.

**Table 1:** Correlation between the expression level of FER1L4 and clinicopathological features in 80 PTC patients

Characteristics	Cases (%)	FER1L4 expression		*P* value*
	n = 80	Low (n = 40)	High (n = 40)	
Age (year)				
≤ 45	43 (53.75)	23	20	0.327
> 45	37 (46.25)	17	20	
Gender				
Male	18 (22.50)	8	10	0.395
Female	62 (77.5)	32	30	
Tumor size (cm)				
≤ 2	69 (86.25)	37	32	0.096
> 2	11 (13.75)	3	8	
Lymph node metastasis				
N0	32 (40.00)	21	11	**0.020**
N1a + N1b	48 (60.00)	19	29	
Extrathyroidal extension				
Yes	12 (15.00)	2	10	**0.013**
No	68 (85.00)	38	30	
TNM stage				
I + II	63 (78.75)	36	27	**0.013**
III + IV	17 (21.25)	4	13	

* Chi-square detection

Significant results were in bold

### FER1L4 promoted cellular growth and motility of PTC

**Fig. 2:**
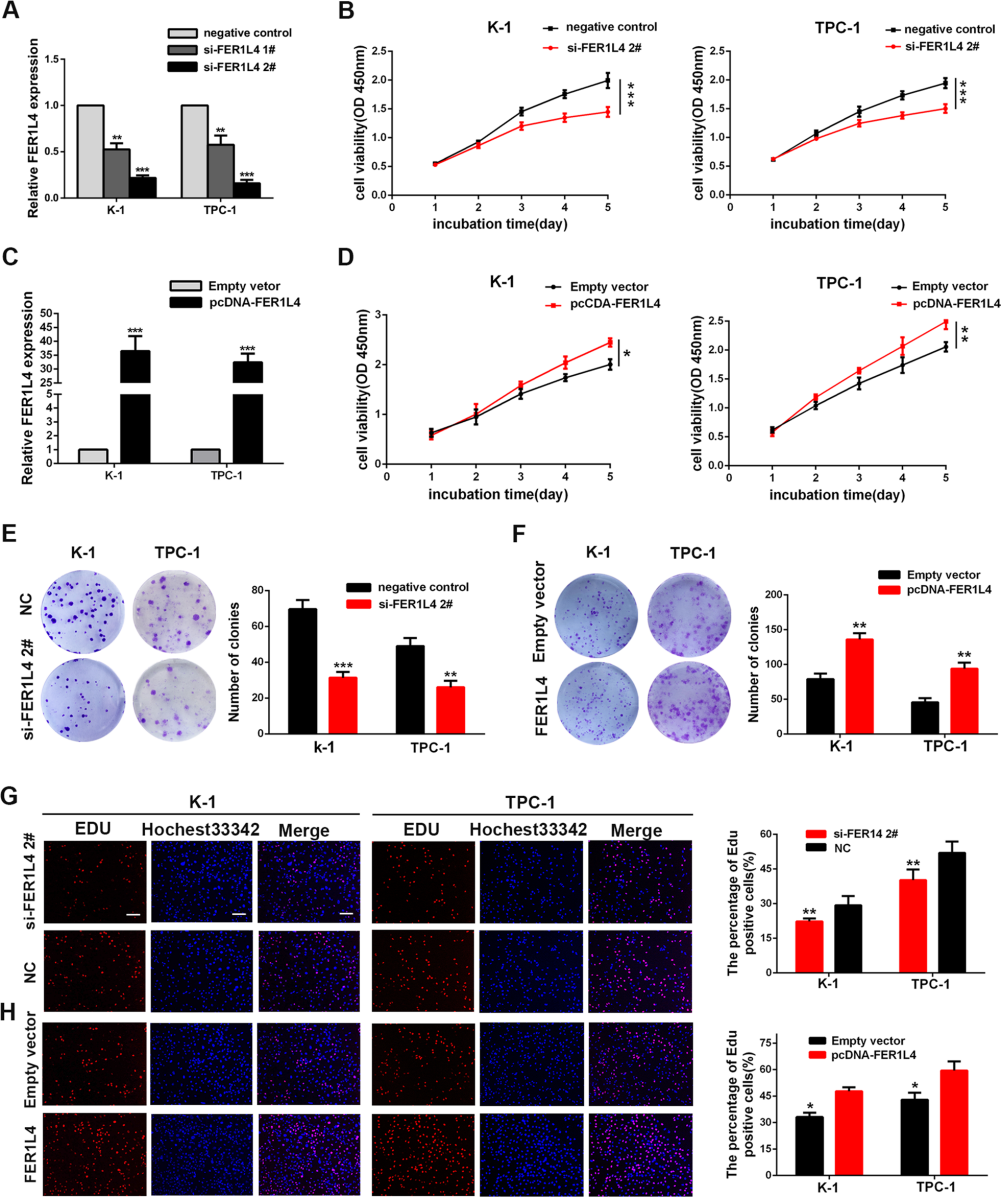
FER1L4 promoted PTC cell proliferation. **A** The expression of FER1L4 was efficiently knocked down using siRNA. **B** Knockdown of FER1L4 inhibited K-1 and TPC-1 cell growth by conducting CCK8 assays. **C** Artificially enhanced expression of FER1L4 in PTC cells. **D** Cellular growth curves of PTC cells treated with FER1L4 vector or empty vector. **E, F** Colony-formation numbers of PTC cells transfected with siRNA targeting FER1L4, and FER1L4 expression vector, compared with negative control. **G, H** The cellular proliferative rate was measured by EdU incorporation assays in PTC cells treated with siRNA targeting FER1L4 and FER1L4 expression vector, compared with negative control. Scale bars = 100 μm. Error bars, mean ± SD. * *P* < 0.05; ** *P* < 0.01; *** *P* < 0.001

**Fig. 3:**
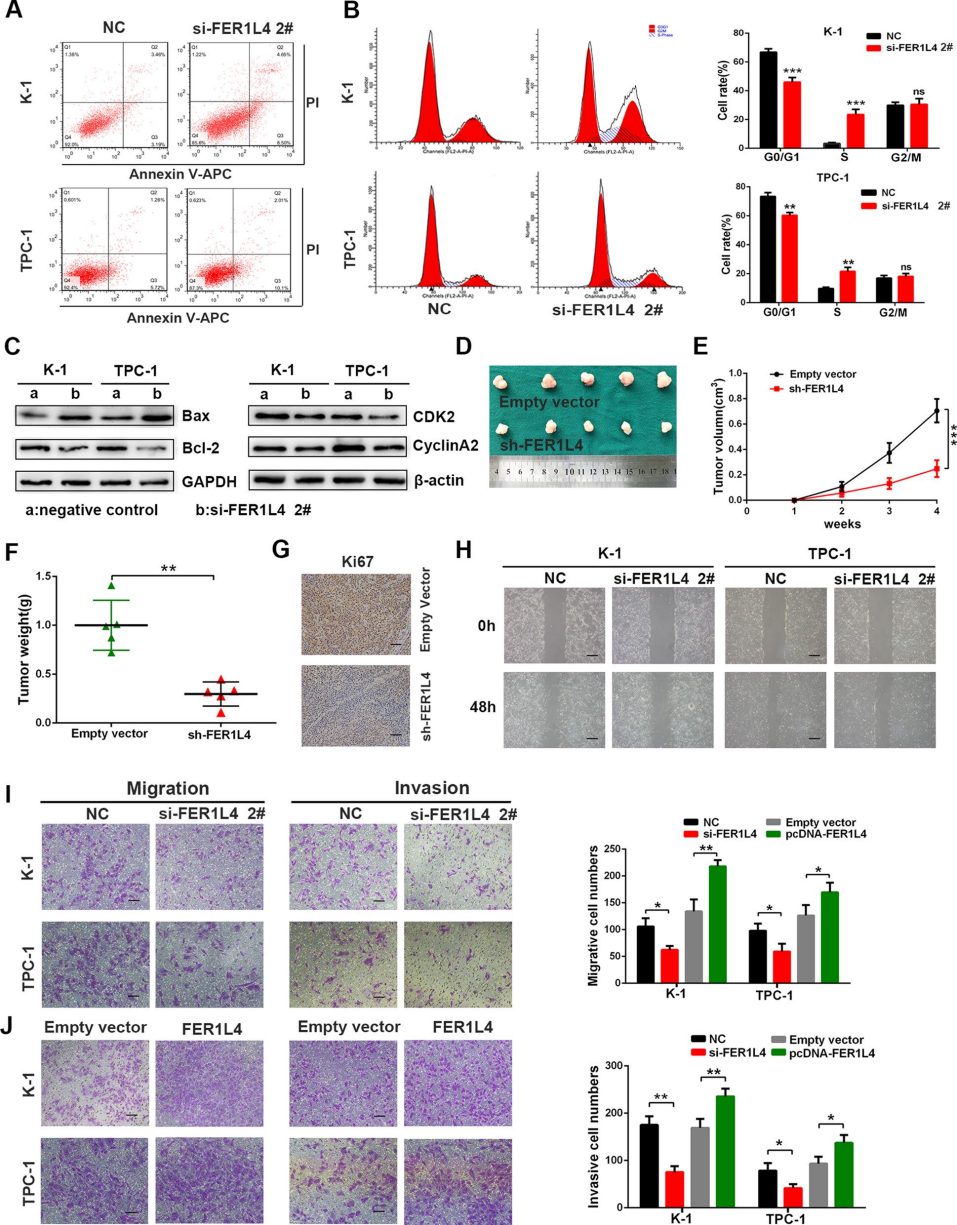
Effects of FER1L4 on cell apoptosis, cell cycle, cell migration, and invasion.** A**, **B** Flow cytometric analysis in apoptotic rate and cell cycle arrest of K-1 and TPC-1 cells transfected with siRNA targeting FER1L4. **C** Expression level of apoptotic markers and cell cycle S phase checkpoint proteins in PTC cells after depletion of FER1L4. **D–F** Tumor growth in mice subcutaneously implanted with FER1L4 silenced TPC-1 cells, and the tumor images, growth curves and weight were shown (n = 5). **G** Representative images of immunohistochemistry staining of Ki67 in implanted tumor sections. Scale bars = 100 μm. **H** Effects of silencing FER1L4 on cellular migration by wound healing assay. **I**, **J** Transwell assays were conducted to investigate the influence of silencing FER1L4 or overexpression of FER1L4 on cellular migration and invasion. Scale bars = 100 μm. Error bars, mean ± SD. * *P* < 0.05; ** *P* < 0.01; *** *P* < 0.001

To elucidate potential functions of FER1L4 in PTC, TPC-1, and K-1 cells were transfected with siRNAs targeting FER1L4 (si-FER1L4 1#, si-FER1L4 2#) (Fig. 2A). However, only siRNA 2# was chosen for the following experiments because of the best efficiency to knock down FER1L4. First, CCK8 assays demonstrated that knockdown of FER1L4 significantly suppressed cellular growth of TPC-1 and K-1 cells (Fig. 2B). Meanwhile, overexpression of FER1L4 showed an enhanced proliferative capacity of PTC cells (Fig. 2C, D). Additionally, colony formation assays indicated that silencing FER1L4 significantly reduced the colony numbers (Fig. 2E), while upregulation of FER1L4 drastically enhanced them (Fig. 2F). Moreover, EdU incorporation assays showed an analogous mode as above, in which EdU positive cell numbers were progressively reduced following depletion of FER1L4 (Fig. 2G), whereas overexpression of FER1L4 resulted in an increase of these cells (Fig. 2H). Importantly, it is successfully identified that decreased apoptosis and cell-cycle promotion are two factors that could contribute to the outgrowth of cancer cells. Herein, we performed flow cytometric assays to examine the role of FER1L4 in these properties. Notably, the knockdown of FER1L4 showed a growing number of apoptotic cells (Fig. 3A; Additional file 2: Fig. S1A). Apart from increased cell apoptosis, FER1L4-silenced PTC cells had a higher percentage of cells in the S phase with detriment to the G0/1 phase than did cells expressed a negative control RNA, which indicated the induction of cell arrest at the S phase after silencing FER1L4 (Fig. 3B). Remarkably, Western blot analysis showed depletion of FER1L4 was accompanied by reduced expression of anti-apoptotic marker like Bcl2, along with enhanced expression of pro-apoptotic protein like Bax. Meanwhile, the expression level of S-phase checkpoint proteins such as CyclinA2 and CDK2 were also decreased in FER1L4-silenced PTC cells (Fig. 3C; Additional file 2: Fig. S1B). In parallel, in order to investigate the function of FER1L4 in vivo, TPC-1 cells transduced with vectors carrying FER1L4 shRNA or scrambled sequence were subcutaneously injected into the flank of 4 weeks nude mice. It showed that depletion of FER1L4 delayed the tumor growth of TPC-1 cells (Fig. 3D), and the tumor size and final tumor weight in implanted tumors significantly decreased in the FER1L4 knockdown group (Fig. 3E, F). Furthermore, immunohistochemistry analysis of orthotopically implanted tumors showed diminished numbers of Ki67-positive cells after silencing FER1L4 (Fig. 3G). Thus, these data indicated that FER1L4 has a critically promoting role in PTC cell growth.

The molecular mechanism behind the capacity for tumor cells to metastasize efficiently is an important topic. Therefore, it is necessary to evaluate whether FER1L4 involves in cellular migration and invasion of PTC. First, wound healing assays showed that knockdown of FER1L4 led to attenuated migration of PTC cells (Fig. 3H; Additional file 2: Fig. S1C). Transwell assays further suggested that loss of FER1L4 resulted in reduced numbers of cells getting through the chamber (Fig. 3I), whereas ectopic expression of FER1L4 showed elevated cell numbers (Fig. 3J). Consequently, our results suggested that FR1L4 essentially involves in the early metastatic process of PTC cells.

### FER1L4 functioned by binding with miR-612 in PTC

**Fig. 4:**
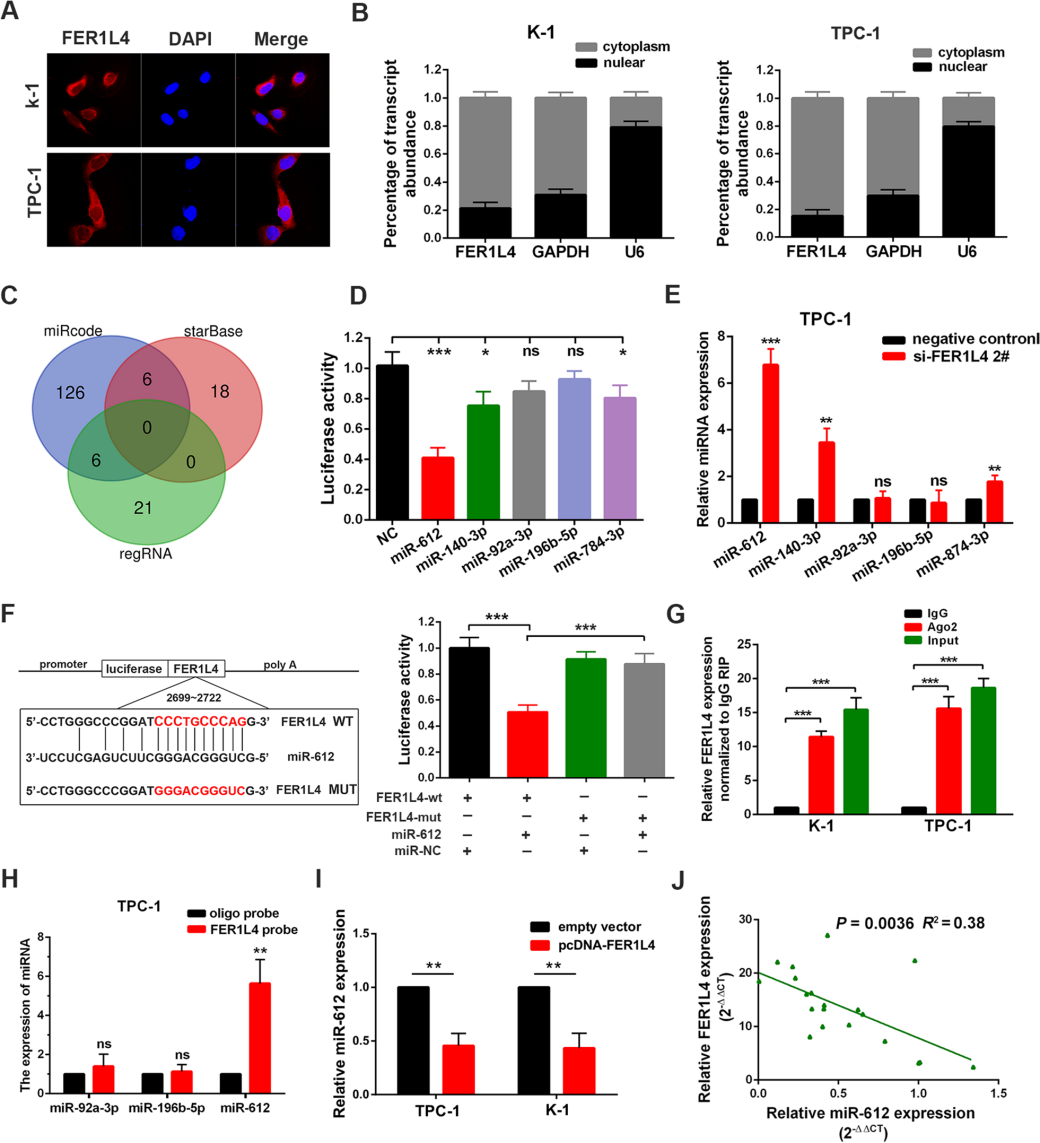
FER1L4 functioned by binding with miR-612 in PTC. **A** Cellular localization of FER1L4 in PTC cells as suggested by FISH assay. Nuclei were stained as blue, and FER1L4 was labeled by Cy3 as red. Scale bars represent 50 μm. **B** Subcellular fractions assays for FER1L4 in K-1 and TPC-1 cells. U6 and GAPDH were used as nuclear and cytoplasmic markers, respectively. **C** MiRNAs with Potential binding sites to FER1L4 were predicted by online tools. **D** Luciferase activity was detected in PTC cells co-transfected with miRNA mimics and luciferase reporter plasmids. **E** The expression level of miRNAs after knockdown of FER1L4 in TPC-1 cells. **F** The binding sequences of miR-612 to FER1L4 were mutated (left panel). And luciferase activity was detected as indicated (right panel). **G** RIP assays were performed in PTC cells using an anti-Ago2 antibody, followed by qRT-PCR analysis for FER1L4. **H** qRT-PCR analysis of miRNAs expression in the RNA complex pulled by FER1L4 probe or oligo probe. **I** Expression of miR-612 in PTC cells transfected with FER1L4 vectors of control. **J** Pearson correlation analysis between miR-612 and FER1L4 in 20 paired PTC tissue samples. Error bars, mean ± SD. * P < 0.05; ** *P* < 0.01; *** *P* < 0.001

Depending on its subcellular location, lncRNA acts in diverse ways to interfere with cellular physiology. In this study, we found FER1L4 mainly located in the cytoplasm of PTC cells by conducting FISH assay and subcellular fractionation assay (Fig. 4A, B). Then, combining with gene expression profiling from the TCGA database, along with predicted results from online tools such as regRNA2.0 [19], miRcode [20], and starbase [21], we selected five miRNAs (miR-612, miR-140-3p, miR-92a-3p, miR-196b-5p, and miR-784-3p) with potential binding sites in FER1L4 sequences (Fig. 4C; Additional file 2: Fig. S2A, B). However, through conducting the luciferase reporter assay, it showed only miR-612, miR-140-3p and miR-784-3p could bind with FER1L4 transcripts (Fig. 4D). Meanwhile, knockdown of FER1L4 also showed upregulated expression of miR-612, miR-140-3p, and miR-784-3p in TPC-1 cells (Fig. 4E). Nevertheless, considering the highest binding affinity between FER1L4 and miR-612, we exclusively chose miR-612 for further studies. As illustrated in Fig. 4F, the binding sequences of miR-612 to FER1L4 were mutated and then fused into a luciferase-reported vector (MUT type). Notably, mutation of miR-612 seed sequence abolished the suppressive effects on FER1L4-driven luciferase activity by miR-612 mimics (Fig. 4F). In addition, the RIP assay further revealed that FER1L4 was successfully immunoprecipitated from cell extract using an anti-Ago2 antibody (Fig. 4G). Moreover, we further measured the binding affinity between FER1L4 and miR-612 by conducting an RNA pull-down assay, followed by qRT-PCR analysis. The results showed FER1L4 probe efficiently binds with miR-612 as compared with the oligo probe (Fig. 4H; Additional file 2: Fig. S2C). Importantly, silencing FER1L4 drastically enhanced the expression of miR-612 in K-1 cells, while overexpression of FER1L4 caused a significant reduction of miR-612 expression (Fig. 4I; Additional file 2: Fig. S2D). On the other hand, a significant inverse correlation between FER1L4 and miR-612 was acquired through analyzing 20 PTC tissue samples (*R*^*2*^ = 0.38, *P* = 0.0036) (Fig. 4J). Taken together, these findings suggested that FRE1L4 could sequester miR-612 away to its targets, therefore involves in PTC tumorigenesis.

### miR-612 was sufficient and necessary for FER1L4

**Fig. 5:**
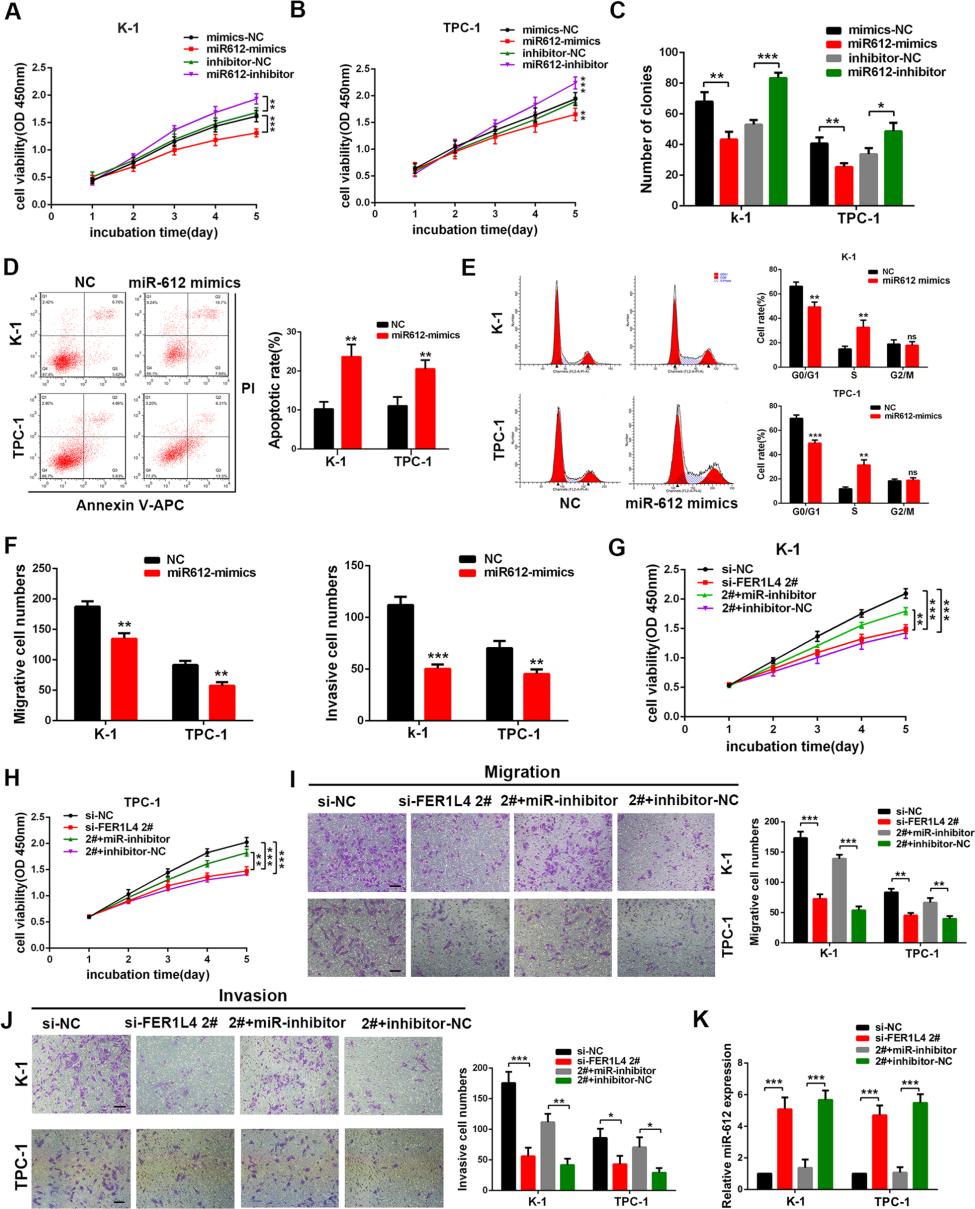
FER1L4 required miR-612 to promote PTC cell growth and invasion.** A**, **B** Detection of cellular proliferation in K-1 and TPC-1 cells as indicated in CCK8 assays. **C** Colony formation assays were performed to evaluate the effect of miR-612 on cellular growth. **D**, **E** Overexpression of miR-612 induced cell apoptosis and cell cycle arrest at the S phase. **F** Ectopic expression of miR-612 inhibited PTC cell migration and invasion in transwell assays. **G**, **H** Cellular growth curves of K-1 and TPC-1 cells co-transfected with miR-612 mimics and FER1L4 siRNA as indicated by CCK8 assays. **I**, **J** Transwell assay was used to determine the migrative ability and invasive ability of PTC cells co-transfected with FER1L4 siRNA and miR-612 inhibitor. **K** The expression of miR-612 was detected by qRT-PCR analysis in PTC cells co-transfected with FER1L4 siRNA and miR-612 inhibitor. Error bars, mean ± SD. * *P* < 0.05; ** *P* < 0.01; *** *P* < 0.001

Although the above data confirmed miR-612 was one of the targets of FER1L4, the function of miR-612 in PTC has never been determined yet. Herein, in order to investigate the effect of miR-612 on PTC cells, the expression of miR-612 was successfully attenuated by miR-612 inhibitor and artificially enhanced by miR-612 mimics (Additional file 2: Fig. S2E). First, overexpression of miR-612 repressed cellular growth (Fig. 5A, B) and colony-formation ability of PTC cells (Fig. 5C; Additional file 2: Fig. S2F), whereas inhibition of miR-612 showed opposite effects. Flow cytometric assays also indicated reintroduction of miR-612 into PTC cells induced cell apoptosis (Fig. 5D) and cell-cycle arrest at S phase (Fig. 5E). Moreover, transwell assays indicated ectopic expression of miR-612 progressively inhibited PTC cell migration and invasion either (Fig. 5F; Additional file 2: Fig. S2G). We next sought to explore whether FER1L4 promotes PTC cell growth and motility via regulating miR-612. PTC cells were co-transfected with FER1L4 siRNA and miR-612 inhibitor, then the resulting cells were subjected to CCK8 assay and transwell assay. It revealed the inhibitory effects on TPC-1 and K-1 cells by knockdown of FER1L4 were nullified by a miRNA-612 inhibitor (Fig. 5G-J). Notably, qRT-PCR analysis verified that FER1L4 siRNA could reverse the suppression on miR-612 expression induced by miR-612 inhibitor, suggesting the binding affinity of FER1L4 to miR-612 was comparable to miR-612 inhibitor (Fig. 5K). As a result, these data unveiled that FER1L4 promoted PTC cell growth and invasion by competitively binding miR-612.

### CDH4 was determined as the target of miR-612

**Fig. 6:**
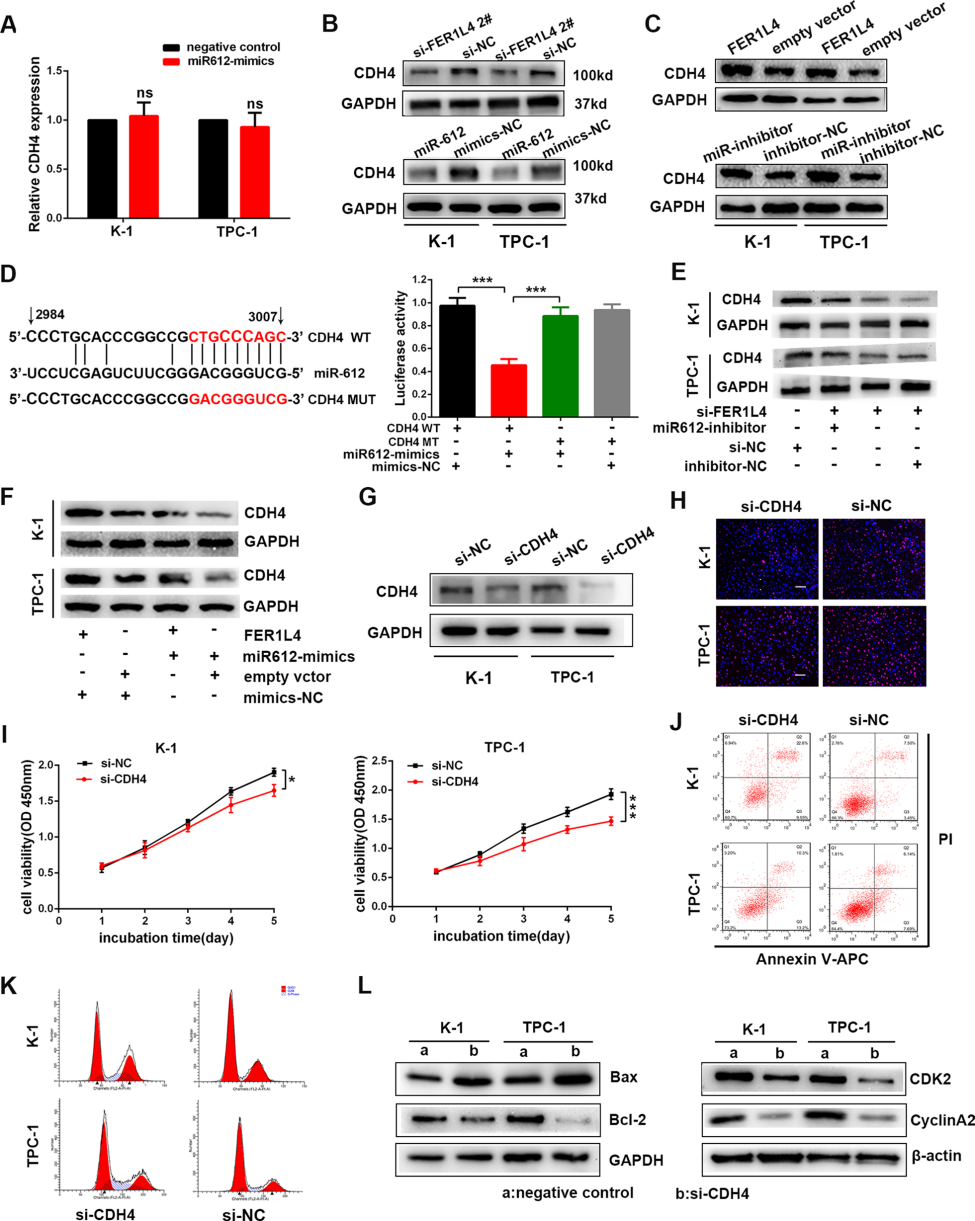
CDH4 was determined as the target of miR-612.** A** qRT-PCR analysis of CDH4 after overexpression of miR-612. **B** Western blot analysis of CDH4 protein level in PTC cells transfected with FER1L4 siRNA, miR-612 mimics, or negative control. **C** Expression of CDH4 in PTC cells transfected with FER1L4 vector, miR-612 inhibitor, or negative control. **D** The binding sites of miR-612 to FER1L4 3′ UTR were mutated (left panel), and then luciferase activity was detected in TPC-1 cells co-transfected with miR-612 mimics and corresponding report vectors as indicated (right panel). **E** Western blot analysis of CDH4 protein level in PTC cells co-transfected with FER1L4 siRNA and miR-612 inhibitor. **F** Detection of CDH4 protein level in PTC cells co-transfected with FER1L4 vector and miR-612 mimics. (**G)** CDH4 expression was efficiently silenced by siRNA. **H, I** Detection of cellular proliferation in PTC cells transfected with siRNA against CDH4 using EdU incorporation assays, and CCK8 assays. Scale bars = 100 μm. **J, K** Flow cytometric analysis of cell apoptosis and cell cycle to cells treated with siRNA targeting CDH4. **L** Relative expression of Bax, Bcl-2, CDK2, and CyclinA2 in CDH4-depleted PTC cells compared with control. Error bars, mean ± SD. * *P* < 0.05; ***, *P* < 0.001

The downstream targets of miR-612 were predicted by online tools such as miRwalk [22], and CDH4 was selected. Intriguingly, the RNA level of CDH4 was not influenced by silencing FER1L4 or overexpression of miR-612 (Fig. 6A; Additional file 2: Fig. S3A). However, the protein level of CDH4 was significantly diminished in PTC cells transfected with siRNA targeting FER1L4 or miR-612 mimics (Fig. 6B; Additional file 2: Fig S3B). On the other hand, PTC cells transfected with FER1L4 vectors or miR-612 inhibitor also exhibited enhanced expression of CDH4 in protein level (Fig. 6C; Additional file 2: Fig. S3C). For subsequent investigation, dual-luciferase reporter assays were performed to validate that miR-612 could bind with CDH4. As presented in Fig. 6D, the binding sites of miR-612 in CDH4 3′ UTR were mutated (MUT-CDH4), and then the mutated 3′ UTR sequence and the wild type 3′ UTR sequence of CDH4 (WT-CDH4) were fused into a plasmid vector respectively. As expected, the luciferase activity was significantly decreased following co-transfection with miR-612 mimics and WT-CDH4 reporter vector, but it was blocked by mutating miR-612 binding sequences (Fig. 6D). Of note, FER1L4 siRNA-induced inhibition of CDH4 expression was rescued by co-transfecting miR-612 inhibitor into PTC cells (Fig. 6E; Additional file 2: Fig. S3D). Meanwhile, miR-612 mimics also successfully reversed FER1L4-mediated promotion on CDH4 expression (Fig. 6F; Additional file 2: Fig. S3E). Thus, based on the above observation, CDH4 was confirmed as a target of miR-612 and therefore was regulated by FER1L4.

### FER1L4/miR-612/CDH4 axis promoted cellular proliferation and invasion of PTC

**Fig. 7:**
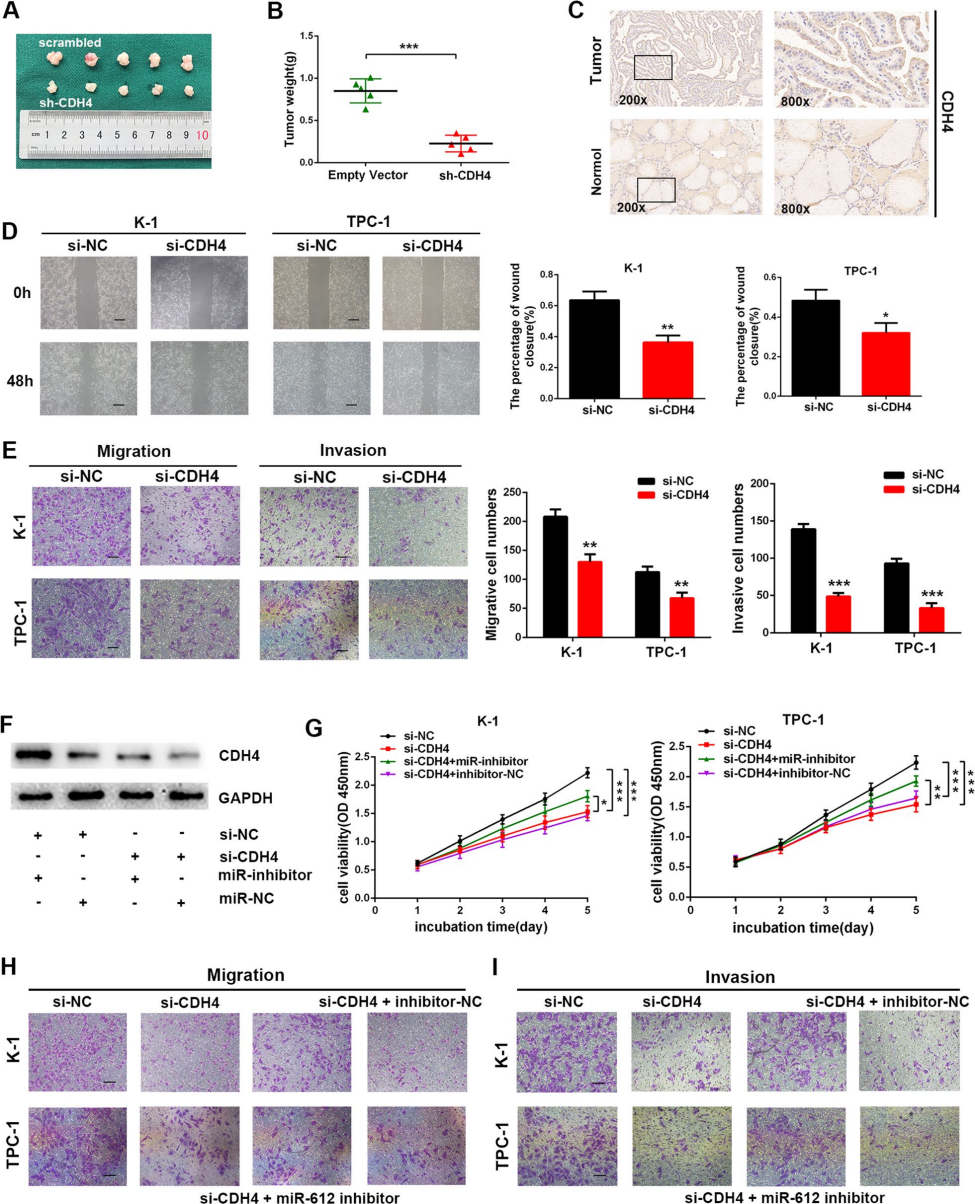
CDH4 promoted PTC cell proliferation and invasion.** A** Implanted tumors in mice were harvested and photographed. **B** The final tumor weight of implanted tumors was calculated. **C** Immunohistochemistry analysis of CDH4 in PTC tissues and adjacent normal tissues. **D**, **E** Knockdown of CDH4 inhibited PTC cell migration and invasion as suggested by wound healing assays and transwell assays. Scale bars = 100 μm. **F** Detection of CDH4 protein level in TPC-1 cells co-transfected with miR-612 inhibitor and CDH4 siRNA. (**G)** Cellular growth curves of PTC cells co-transfected with miR-612 inhibitor and CDH4 siRNA determined by CCK8 assays. **H**, **I** Rescue effects of miR-612 inhibitor on CDH4 siRNA-mediated inhibition of cellular migration and invasion. Error bars, mean ± SD. * *P* < 0.05; ** *P* < 0.01; *** *P* < 0.001

It was clear that the expression of CDH4 was upregulated in TC tissues and cell lines (Additional file 2: Fig. S4A, B). To delineate further the functional significances of CDH4 in PTC cell proliferation and invasion, the expression of CDH4 was efficiently knocked down in K-1 and TPC-1 cells (Fig. 6G; Additional file 2: Fig. S4C, D). First, loss of CDH4 repressed cellular proliferation as presented by EdU incorporation assay (Fig. 6H; Additional file 2: Fig. S4E) and CCK8 assay (Fig. 6I). Furthermore, silencing CDH4 also displayed significant induction of apoptosis accompanied by elevated Bax expression and reduced Bcl2 expression, as well as cell cycle arrest at S phase with reduced proteins level of CDK2 and cyclinA2 (Fig. 6J-L; Additional file 2: Fig. S4F-H). Importantly, subcutaneous tumor-formation experiments revealed that knockdown of CDH4 significantly inhibited the formation of subcutaneous tumors of PTC cells (Fig. 7A, B; Additional file 2: Fig. S4I). In addition, IHC analysis of CDH4 also showed upregulated expression of CDH4 in PTC tissue samples (Fig. 7C). Of note, wound healing assays also uncovered a reduction of cell migrative ability in CDH4-depleted cells (Fig. 7D). Consistently, transwell assays displayed a sharp decline in cell numbers getting through the chamber by knockdown of CDH4 (Fig. 7E). Together, these experiments support an oncogenic role for CDH4 in promoting PTC cell proliferation and invasion.

Subsequently, we performed several functional experiments in CDH4-depleted cells with simultaneous knockdown of miR-612. First, co-transfection with a miR-612 inhibitor in PTC cells resulted in a reversal of CDH4 siRNA-induced repression on the CDH4 protein level (Fig. 7F). Then, we further examined the effects on cellular proliferation and invasion. As indicated by CCK8 assays, CDH4 siRNA-mediated suppression in cell growth was nullified by artificially lowering the expression of miR-612 (Fig. 7G). And an analogous mode was detected in transwell assays (Fig. 7H, I; Additional file 2: Fig. S4J). In conclusion, these results suggested there may exist an axis between FER1L4, miR-612, and CDH4 which involves the tumorigenesis and aggressiveness of papillary thyroid cancer.

## Discussion

The development of thyroid cancer involves multiple genetic and epigenetic alterations such as point mutation of the BRAF and RAS genes which seem to be linked to specific etiologic factors like exposure to ionizing radiation and chemical mutagenesis [23]. However, understanding the detailed molecular mechanisms how thyroid cancer development is still a major scientific challenge. In recent decades, technical advances in genome editing and high-throughput sequencing have promoted the deep research of non-coding genomes [24]. Notably, long noncoding RNAs, the main components of non-coding genes, are considered to be essentially implicated in the process of tumor initiation or progression including thyroid cancer [25]. Fer-1 like family member 4 is a lncRNA with 6.7 kb length, located in chromosome 20 q11.22. However, intriguingly, plenty of literature documents context-dependent phenotypes of FER1L4 in human cancers. It was previously reported that FER1L4 served as a tumor suppressor in colon cancer [26] and gastric cancer [27], while it showed an oncogenic role in glioma [28]. In a human pan‐cancer analysis, it suggested FER1L4 may act as an oncogenic driver in cancers [29]. Thus, importantly, the current study unraveled a new facet of FER1L4 which accelerates PTC cell proliferation, migration, and invasion. In agreement, upregulated expression of FER1L4 in PTC tissues was closely related to advanced clinicopathological features such as lymph node metastasis, extrathyroidal extension, and advanced TNM stage. Accordingly, FER1L4 was considered as an oncogene in PTC.

In our study, the confirmation of FER1L4 localized in cytoplasm given us a reason to hypothesize that FER1L4 may influence the progression of PTC through the mechanism model of ceRNA. Subsequently, miR-612 was validated to directly bind with FER1L4 in PTC cells. MiR-612 has been proved to suppress tumor growth and metastases in many tumors. For example, miR-612 negatively controlled the formation of invadopodia, matrix degradation, and metastasis in hepatocellular carcinoma by involving HADHA-mediated lipid reprogramming [30]. Nevertheless, the function of miR-612 in PTC was never described before. Herein, in the present study, miR-612 was also successfully identified as a tumor suppressor which inhibits cellular proliferation, migration, and invasion of PTC. Meanwhile, functional experiments also suggested FER1L4-induced promotion on PTC cells was mediated by negatively regulating miR-612. Hence, our findings uncovered that FER1L4 exerts its oncogenic behavior in PTC via sponging miR-612, at least in a part. Of note, the subcellular fraction assays also suggested that FER1L4 partially exists in the nucleus of PTC cells. Thus, exploration of the detailed mechanisms of how FER1L4 carries out its functions in the nucleus is quite necessary. For instance, HOTAIR (HOX transcript antisense intergenic RNA), a well-known lncRNA localized both in the cytoplasm and nucleus, could modulate chromatin accessibility by serving as a scaffold for the PRC2 subunit EZH2, or act also at the posttranscriptional level by sponging several miRNAs[31, 32]. In a manner similar to HOTAIR, FER1L4 may also interact with chromatin and then recruit protein complexes to remodel chromatin states, thus causing manifest changes in gene expression and gene regulatory network[33, 34]. However, it needs further experimental confirmation.

Here, CDH4 was proved to be the target of miR-612. However, FER1L4 or miR-612 only influences the expression of CDH4 at the protein level, indicating miR-612 may repress the translation of CDH4 and then attenuates its expression [35]. Deregulation of CDH4 has been implicated in several human cancers. Dependent on cell context, in most epithelial cancers like breast cancer [36], colorectal and gastric cancer [37], CDH4 is epigenetically silenced by promoter hypermethylation and commonly acts as a tumor suppressor. Nevertheless, some researches also support an oncogenic function of CDH4, such as in high-grade gliomas [17] and osteosarcoma [38]. Although there are discrepancies in the functions of CDH4 in human cancers, our study identified and suggested an oncogenic role of CDH4 in PTC. Consistent with its function in cell–cell adhesion, the immunohistochemical analysis indicated that CDH4 showed crisp membrane localization in PTC tissues. In particular, Gene Set Enrichment Analysis (GSEA) of CDH4 using gene expression data from the TCGA database also validated that CDH4 is involved in processes such as adherens junction and extracellular matrix-receptor interaction in thyroid cancer (Additional file 2: Fig. S5A, B) [39]. On the other hand, FER1L4 was also characterized with association to gene set of cell adhesion molecules (Additional file 2: Fig. S5C). However, correlation analysis of thyroid cancer cohort from TCGA database suggested FER1L4 was positively correlated with P-cadherin (*R*^*2*^ = 0.2513, *P* < 0.001), but not E-cadherin, N-cadherin, or CDH4 (Additional file 2: Fig. S5E-H), which was consistent with the qRT-PCR results (Additional file 2: Fig. S3A). Intriguingly, the expression of CDH4 was also positively correlated with P-cadherin using the TCGA database (*R*^*2*^ = 0.1156, *P* < 0.001; Additional file 2: Fig. S5K), indicating that FER1L4 may regulate the expression of P-cadherin via governing CDH4 or vice versa [40]. However, it needs further experimental confirmation. Meanwhile, exploring the mechanism of CDH4 in PTC progression is an important topic either. It was reported CDH4 is necessary to the activation of ERK and p27 [17], and also of c-Jun [38]. GSEA analysis suggested CDH4 was positively correlated with the Wnt/β-catenin pathway, which may indicate the underlying mechanism of CDH4 (Additional file 2: Fig. S5D). However, the underlying mechanism of CDH4 in PTC carcinogenesis requires thorough exploration. Of note, the distinct features of FER1L4 and CDH4 in cancer may be caused by the heterogeneity of tumor [41], suggesting the great potential for FER1L4 and CDH4 as diagnostic biomarkers of PTC.

## Conclusions

Taken together, FER1L4 promoted PTC development via working as a ceRNA for miR-612 to attenuate the repressive effect on CDH4. These results provided a better understanding of the role of lncRNA in PTC progression, as well as suggested potential therapeutic and diagnostic targets for PTC.

## Supplementary Information

**Additional file 1: Table S1.** Primers used for PCR. **Table S2.** siRNA and shRNA used in this study. **Table S3.** Antibodies used for Western blot.

**Additional file 2:** **Figure S1.** Quantitative analysis of the effect of FER1L4 on cell apoptosis and cell cycle. **Figure S2.** FER1L4-targeting miR-612 suppressed PTC cell migration and invasion. **Figure S3.** Quantitative analysis of the effect of FER1L4 and miR-612 on CDH4 expression. **Figure S4.** Quantitative analysis of the oncogenic effects of CDH4 on PTC cells. **Figure S5.** GSEA analysis of FER1L4 and CDH4, and correlation analysis between cadherins and FER1L4.

## Data Availability

The original contributions presented in the study are included in the article/Supplementary Material, further inquiries can be directed to the corresponding author.

## Abbreviations

PTC: Papillary thyroid cancer
TC: Thyroid cancer
DTC: Differentiated thyroid cancer
LncRNA: Long noncoding RNA
FER1L4: Fer-1 like family member 4
CDH4: Cadherin 4
CeRNA: Competing endogenous RNA
TNM: Tumor-node-metastasis
qRT-PCR: Quantitative real-time PCR
CCK8: Cell counting kit 8
IHC: Immunohistochemical
RIP: RNA immunoprecipitation
TCGA: The Cancer Genome Atlas
GSEA: Gene Set Enrichment Analysis
ECM: Extracellular matrix
EMT: Epithelial–mesenchymal transition
SiRNA: Small interfering RNA

## References

[1] Bray F, Ferlay J, Soerjomataram I, Siegel RL, Torre LA, Jemal A (2018). Global cancer statistics 2018: GLOBOCAN estimates of incidence and mortality worldwide for 36 cancers in 185 countries. CA Cancer J Clin.

[2] Haugen BR, Alexander EK, Bible KC, Doherty GM, Mandel SJ, Nikiforov YE, Pacini F, Randolph GW, Sawka AM, Schlumberger M (2016). 2015 American Thyroid Association Management Guidelines for Adult Patients with Thyroid Nodules and Differentiated Thyroid Cancer: The American Thyroid Association Guidelines Task Force on Thyroid Nodules and Differentiated Thyroid Cancer. Thyroid.

[3] Lim H, Devesa SS, Sosa JA, Check D, Kitahara CM (2017). Trends in thyroid cancer incidence and mortality in the United States, 1974–2013. JAMA.

[4] Links TP, van Tol KM, Jager PL, Plukker JT, Piers DA, Boezen HM, Dullaart RP, de Vries EG, Sluiter WJ (2005). Life expectancy in differentiated thyroid cancer: a novel approach to survival analysis. Endocr Relat Cancer.

[5] Kreissl MC, Janssen MJR, Nagarajah J (2019). Current treatment strategies in metastasized differentiated thyroid cancer. J Nucl Med.

[6] Geisler S, Coller J (2013). RNA in unexpected places: long non-coding RNA functions in diverse cellular contexts. Nat Rev Mol Cell Biol.

[7] Bhan A, Soleimani M, Mandal SS (2017). Long Noncoding RNA and Cancer: A New Paradigm. Cancer Res.

[8] Boon RA, Jae N, Holdt L, Dimmeler S (2016). Long noncoding RNAs: from clinical genetics to therapeutic targets?. J Am Coll Cardiol.

[9] Kang CL, Qi B, Cai QQ, Fu LS, Yang Y, Tang C, Zhu P, Chen QW, Pan J, Chen MH, Wu XZ (2019). LncRNA AY promotes hepatocellular carcinoma metastasis by stimulating ITGAV transcription. Theranostics.

[10] Liang Y, Song X, Li Y, Chen B, Zhao W, Wang L, Zhang H, Liu Y, Han D, Zhang N (2020). LncRNA BCRT1 promotes breast cancer progression by targeting miR-1303/PTBP3 axis. Mol Cancer.

[11] Chen LL (2016). Linking Long Noncoding RNA Localization and Function. Trends Biochem Sci.

[12] Yao RW, Wang Y, Chen LL (2019). Cellular functions of long noncoding RNAs. Nat Cell Biol.

[13] Qin Y, Sun W, Wang Z, Dong W, He L, Zhang T, Shao L, Zhang H (2020). ATF2-Induced lncRNA GAS8-AS1 promotes autophagy of thyroid cancer cells by targeting the miR-187-3p/ATG5 and miR-1343-3p/ATG7 Axes. Mol Ther Nucleic Acids.

[14] Colas-Algora N, Millan J (2019). How many cadherins do human endothelial cells express?. Cell Mol Life Sci.

[15] Kaszak I, Witkowska-Pilaszewicz O, Niewiadomska Z, Dworecka-Kaszak B, Ngosa Toka F, Jurka P (2020). Role of cadherins in cancer-a review. Int J Mol Sci.

[16] Kourtidis A, Lu R, Pence LJ, Anastasiadis PZ (2017). A central role for cadherin signaling in cancer. Exp Cell Res.

[17] Appolloni I, Barilari M, Caviglia S, Gambini E, Reisoli E, Malatesta P (2015). A cadherin switch underlies malignancy in high-grade gliomas. Oncogene.

[18] Zhuang X, Tong H, Ding Y, Wu L, Cai J, Si Y, Zhang H, Shen M (2019). Long noncoding RNA ABHD11-AS1 functions as a competing endogenous RNA to regulate papillary thyroid cancer progression by miR-199a-5p/SLC1A5 axis. Cell Death Dis.

[19] Huang HY, Chien CH, Jen KH, Huang HD (2006). RegRNA: an integrated web server for identifying regulatory RNA motifs and elements. Nucleic Acids Res.

[20] Jeggari A, Marks DS, Larsson E (2012). miRcode: a map of putative microRNA target sites in the long non-coding transcriptome. Bioinformatics.

[21] Li JH, Liu S, Zhou H, Qu LH, Yang JH (2014). starBase v2.0: decoding miRNA-ceRNA, miRNA-ncRNA and protein-RNA interaction networks from large-scale CLIP-Seq data. Nucleic Acids Res.

[22] Sticht C, De La Torre C, Parveen A, Gretz N (2018). miRWalk: An online resource for prediction of microRNA binding sites. PLoS ONE.

[23] Nikiforov YE, Nikiforova MN (2011). Molecular genetics and diagnosis of thyroid cancer. Nat Rev Endocrinol.

[24] Djebali S, Davis CA, Merkel A, Dobin A, Lassmann T, Mortazavi A, Tanzer A, Lagarde J, Lin W, Schlesinger F (2012). Landscape of transcription in human cells. Nature.

[25] Sedaghati M, Kebebew E (2019). Long noncoding RNAs in thyroid cancer. Curr Opin Endocrinol Diabetes Obes.

[26] Yue B, Sun B, Liu C, Zhao S, Zhang D, Yu F, Yan D (2015). Long non-coding RNA Fer-1-like protein 4 suppresses oncogenesis and exhibits prognostic value by associating with miR-106a-5p in colon cancer. Cancer Sci.

[27] Xu J, Li N, Deng W, Luo S (2020). Long noncoding RNA FER1L4 suppresses proliferation, invasion, migration and lymphatic metastasis of gastric cancer cells through inhibiting the Hippo-YAP signaling pathway. Am J Transl Res.

[28] Xia L, Nie D, Wang G, Sun C, Chen G (2019). FER1L4/miR-372/E2F1 works as a ceRNA system to regulate the proliferation and cell cycle of glioma cells. J Cell Mol Med.

[29] You Z, Ge A, Pang D, Zhao Y, Xu S (2020). Long noncoding RNA FER1L4 acts as an oncogenic driver in human pan-cancer. J Cell Physiol.

[30] Liu Y, Lu LL, Wen D, Liu DL, Dong LL, Gao DM, Bian XY, Zhou J, Fan J, Wu WZ (2020). MiR-612 regulates invadopodia of hepatocellular carcinoma by HADHA-mediated lipid reprogramming. J Hematol Oncol.

[31] Battistelli C, Garbo S, Riccioni V, Montaldo C, Santangelo L, Vandelli A, Strippoli R, Tartaglia GG, Tripodi M, Cicchini C (2021). Design and functional validation of a mutant variant of the LncRNA HOTAIR to counteract snail function in epithelial-to-mesenchymal transition. Cancer Res.

[32] Liu B, Liu Q, Pan S, Huang Y, Qi Y, Li S, Xiao Y, Jia L (2019). The HOTAIR/miR-214/ST6GAL1 crosstalk modulates colorectal cancer procession through mediating sialylated c-Met via JAK2/STAT3 cascade. J Exp Clin Cancer Res.

[33] Zhang G, Lan Y, Xie A, Shi J, Zhao H, Xu L, Zhu S, Luo T, Zhao T, Xiao Y, Li X (2019). Comprehensive analysis of long noncoding RNA (lncRNA)-chromatin interactions reveals lncRNA functions dependent on binding diverse regulatory elements. J Biol Chem.

[34] Dong A, Preusch CB, So WK, Lin K, Luan S, Yi R, Wong JW, Wu Z, Cheung TH (2020). A long noncoding RNA, LncMyoD, modulates chromatin accessibility to regulate muscle stem cell myogenic lineage progression. Proc Natl Acad Sci USA.

[35] Lee YS, Dutta A (2009). MicroRNAs in cancer. Annu Rev Pathol.

[36] Agiostratidou G, Li M, Suyama K, Badano I, Keren R, Chung S, Anzovino A, Hulit J, Qian B, Bouzahzah B (2009). Loss of retinal cadherin facilitates mammary tumor progression and metastasis. Cancer Res.

[37] Miotto E, Sabbioni S, Veronese A, Calin GA, Gullini S, Liboni A, Gramantieri L, Bolondi L, Ferrazzi E, Gafà R (2004). Frequent aberrant methylation of the CDH4 gene promoter in human colorectal and gastric cancer. Cancer Res.

[38] Tang Q, Lu J, Zou C, Shao Y, Chen Y, Narala S, Fang H, Xu H, Wang J, Shen J, Khokha R (2018). CDH4 is a novel determinant of osteosarcoma tumorigenesis and metastasis. Oncogene.

[39] Mège RM, Ishiyama N (2017). Integration of cadherin adhesion and cytoskeleton at adherens junctions. Cold Spring Harb Perspect Biol.

[40] Martinez-Garay I, Gil-Sanz C, Franco SJ, Espinosa A, Molnar Z, Mueller U (2016). Cadherin 2/4 signaling via PTP1B and catenins is crucial for nucleokinesis during radial neuronal migration in the neocortex. Development.

[41] Meacham CE, Morrison SJ (2013). Tumour heterogeneity and cancer cell plasticity. Nature.

