# Supplementary material for: Long noncoding RNA FER1L4 promotes the malignant processes of papillary thyroid cancer by targeting the miR-612/ Cadherin 4 axis
Source: Cancer Cell Int. 2021 Jul 21;21:392. doi: 10.1186/s12935-021-02097-2 (PMC8296567; doi:10.1186/s12935-021-02097-2)
Supplement: Supplementary file 1 — Additional file 1: Table S1. Primers used for PCR. Table S2. siRNA and shRNA used in this study. Table S3. Antibodies used for Western blot. [file 12935_2021_2097_MOESM1_ESM.docx]

**Title:** Long noncoding RNA FER1L4 promotes papillary thyroid cancer progression by targeting miR-612/CDH4 axis

**Additional file 1: Supplementary Tables 1-3**

**Table S1 Primers used for qRT-PCR.**

| **RNA** | **5'to 3'** |
| --- | --- |
| FER1L4 | Forward CAACCTGAACGCCATCAACATC  Reverse GGCAAGTCCACTGTCAGATG |
| GAPDH | Forward TGCACCACCAACTGCTTAGC  Reverse GGCATGGACTGTGGTCATGAG |
| β-actin | Forward GCATCGTCACCAACTGGGAC  Reverse ACCTGG CCGTCAGGCAGCTC |
| CDH4 | Forward CAACCTGAACGCCATCAACATC  Reverse CGCAAGCTGAGTTGGGCATAG |
| Universal reverse primer | GCGAGCACAGAATTAATACGAC |
| U6 | Forward CTCGCTTCGGCAGCACA  Universal reverse primer |
| miR-612 | Forward GCAGGGCTTCTGAGCTCCTTAA  Universal reverse primer |
| miR-140-3P | Forward TACCACAGGGTAGAACCACGG  Universal reverse primer |
| miR-92a-3p | Forward TATTGCACTTGTCCCGGCCTGT  Universal reverse primer |
| miR-196b-5p | Forward TAGGTAGTTTCCTGTTGTTGGG  Universal reverse primer |
| miR-874-3p | Forward CUGCCCUGGCCCGAGGGACCGA  Universal reverse primer |

**Table S2 siRNA and shRNA used in this study.**

| **siRNA** | **5'to 3'** |
| --- | --- |
| si-FER1L4 1# | sense CAGGACAGCUUCGAGUUAATT  antisense UUAACUCGAAGCUGUCCUGTT |
| si-FER1L4 2# | sense CAGGACAGCUUCGAGUUAATT  antisense UUAACUCGAAGCUGUCCUGTT |
| siRNA-negative  control | sense UUCUCCGAACGUGUCACGUTT  antisense ACGUGACACGUUCGGAGAATT |
| sh-FER1L4 | GGAAGTCTTTCTTACCCATGT |
| miR-612 mimics | sense GCUGGGCAGGGCUUCUGAGCUCCUU  antisense GGAGCUCAGAAGCCCUGCCCAGCUU |
| miR-612 mimics negative control | sense UUCUCCGAACGU GUC ACGUTT  antisense ACGUGACACGUUCGGAGAATT |
| miR-612 inhibitor | AAGGAGCUCAGAAGCCCUGCCCAGC |
| miR-612 inhibitor  negative control | CAGUACUUUUGUGUAGUACAA |
| si-CDH4 | sense CCACGUUUUCAGCUGUGGATT  antisense UCCACAGCUGAAAACGUGGTT |

| **antibody** | **company/provider (Dilution ratio)** |
| --- | --- |
| GAPDH | Proteintech, Wuhan, China (1:10000) |
| β-actin | Proteintech, Wuhan, China (1:10000) |
| anti-CDH4 | Abclonal, Wuhan, China (1:1000) |
| anti-CyclinA2 | Proteintech, Wuhan, China (1:2000) |
| anti-CDK2 | Proteintech, Wuhan, China (1:2000) |
| anti-Bax | Proteintech, Wuhan, China (1:2000) |
| anti-Bcl-2 | Proteintech, Wuhan, China (1:2000) |
| anti -Ago2 | Abcam, CA, MA, USA (1:1000) |

**Table S3 Antibodies used for Western blot.**
