# Supplementary material for: Long noncoding RNA FER1L4 promotes the malignant processes of papillary thyroid cancer by targeting the miR-612/ Cadherin 4 axis
Source: Cancer Cell Int. 2021 Jul 21;21:392. doi: 10.1186/s12935-021-02097-2 (PMC8296567; doi:10.1186/s12935-021-02097-2)
Supplement: Supplementary file 2 — Additional file 2: Figure S1. Quantitative analysis of the effect of FER1L4 on cell apoptosis and cell cycle. Figure S2. FER1L4-targeting miR-612 suppressed PTC cell migration and invasion. Figure S3. Quantitative analysis of the effect of FER1L4 and miR-612 on CDH4 expression. Figure S4. Quantitative analysis of the oncogenic effects of CDH4 on PTC cells. Figure S5. GSEA analysis of FER1L4 and CDH4, and correlation analysis between cadherins and FER1L4. [file 12935_2021_2097_MOESM2_ESM.docx]

**Title:** Long noncoding RNA FER1L4 promotes papillary thyroid cancer progression by targeting the miR-612/CDH4 axis

**Additional file 2: Supplementary Figures S1-5**

**Figure S1 Quantitative analysis of the effect of FER1L4 on cell apoptosis and cell cycle.**


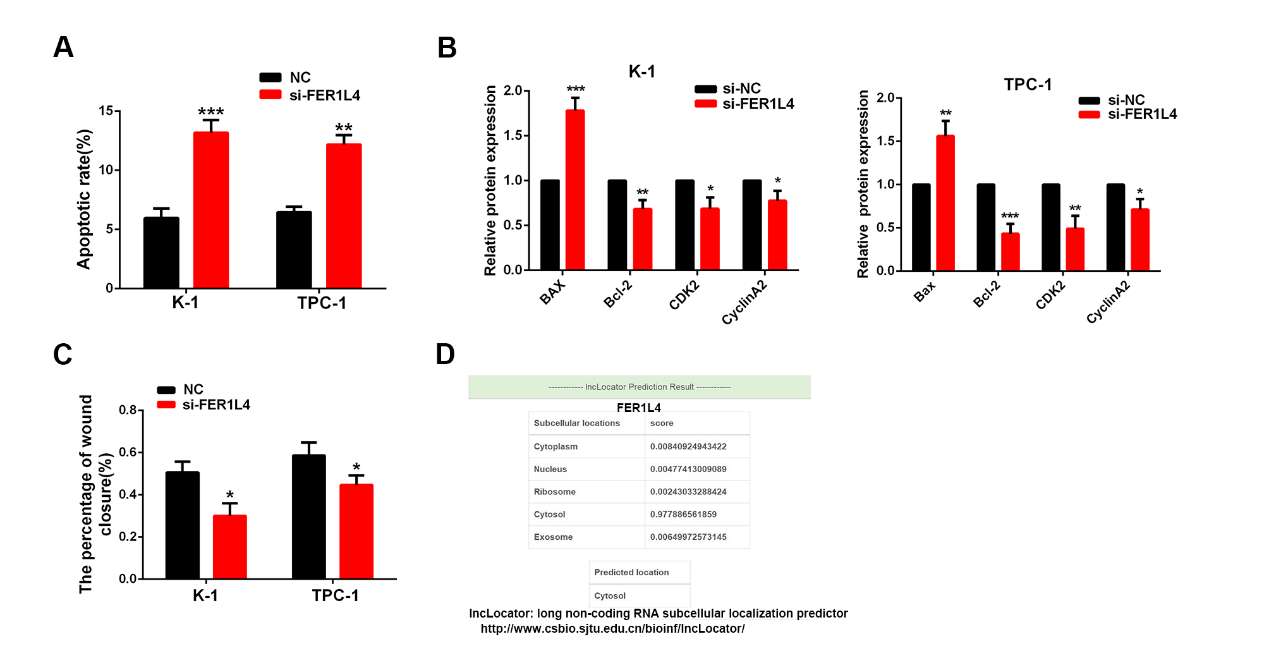


**Figure S1** **Quantitative analysis of the effect of FER1L4 on cell apoptosis and cell cycle. (A)** Knockdown of FER1L4 induced cell apoptosis. (**B)** Relative protein expression in K-1 and TPC-1 cells transfected with FER1L4 targeting siRNA or control. (**C)** Effects on cell migrative ability suggested by wound healing assays in FER1L4-depleted K-1 and TPC-1 cells. (**D)** Online prediction of the subcellular localization to FER1L4 by LncLocator. Error bars, mean ± SD. * *P* < 0.05; ** *P* < 0.01; *** *P* <0.001.

**Figure S2 FER1L4-targeting miR-612 suppressed PTC cell migration and invasion.**


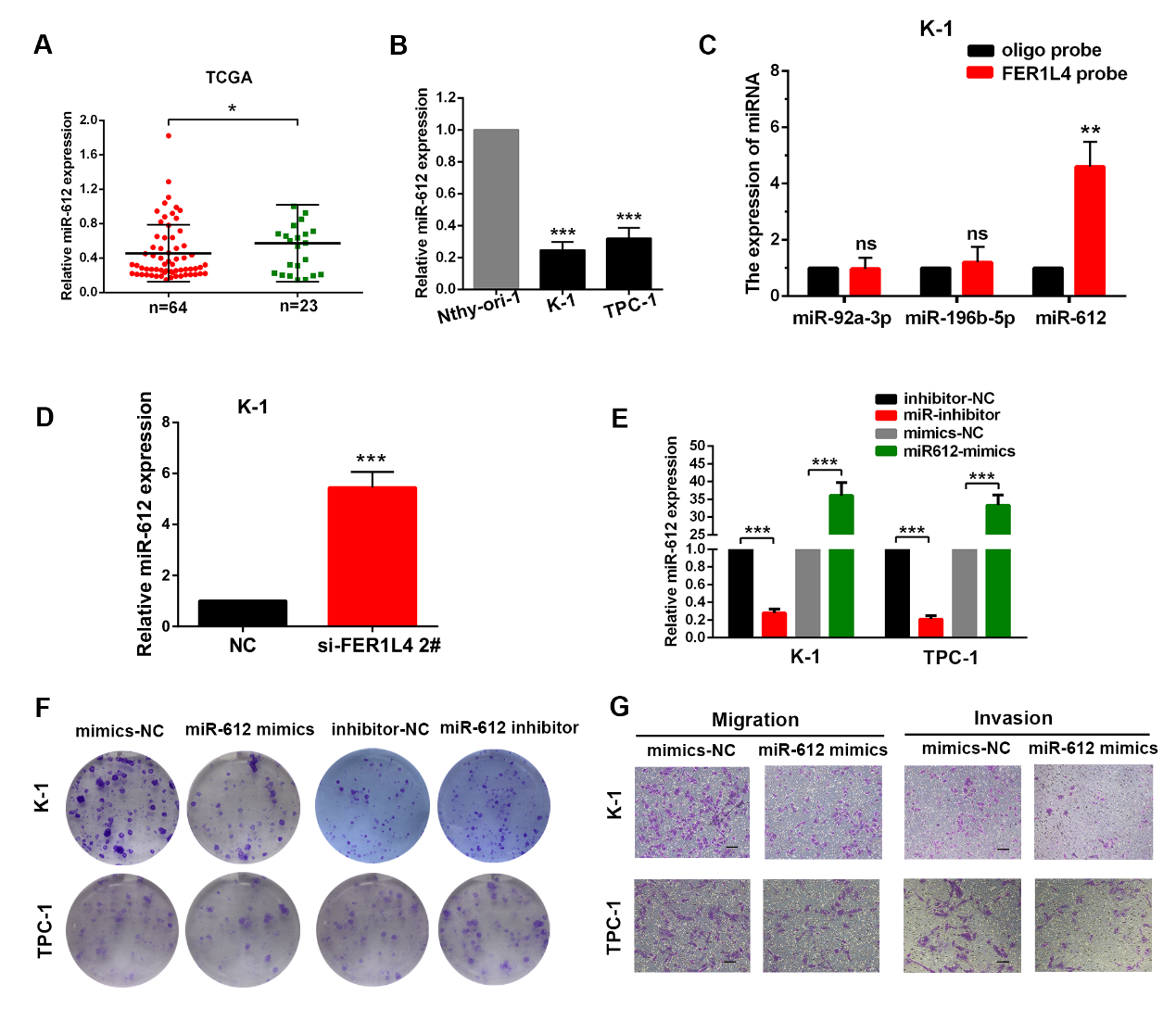


**Figure S2** **FER1L4-targeting miR-612 suppressed PTC cell migration and invasion. (A)** The expression level of miR-612 in PTC tissues compared with normal tissues from TCGA database. **(B)** The expression level of miR-612 in K-1 and TPC-1 cells compared with Nthy-ori-1. (**C)** qRT-PCR analysis of miRNAs expression in the RNA complex pulled by FER1L4 probe or oligo probe. (**D**) The expression of miR-612 in K-1 cells treated with siRNA targeting FER1L4. (**E**) Expression level of miR-612 in cells treated with miR-612 inhibitor and mimics, or negative control. (**F)** Effects of miR-612 on cellular proliferation of K-1 and TPC-1 cells suggested by colony formation assays. (**G)** Effects of miR-612 on cellular migration and invasion of K-1 and TPC-1 cells suggested by transwell assays. Scale bar = 100 um. Error bars, mean ± SD. * *P* < 0.05; *** *P* <0.001.

**Figure S3** **Quantitative analysis of the effect of FER1L4 and miR-612 on CDH4 expression.**


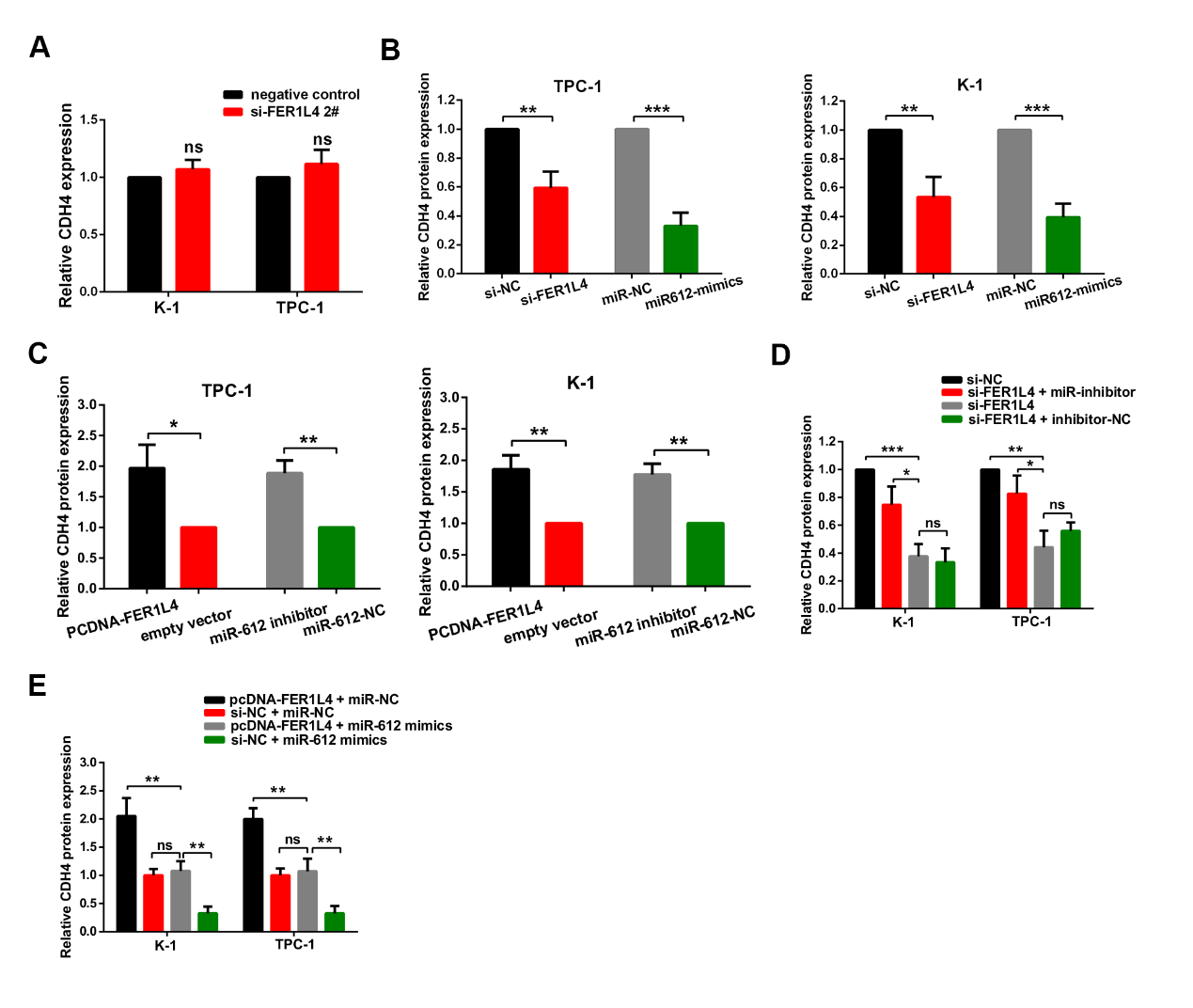


**Figure S3 Quantitative analysis of the effect of FER1L4 and miR-612 on CDH4 expression. (A)** Relative CDH4 RNA level in PTC cells treated with FER1L4 targeting siRNA. (**B)** Quantitative analysis of CDH4 protein level in TPC-1 and K-1 cells transfected with siRNA targeting FER1L4, miR-612 mimics, or negative control. (**C)** Western blot analysis of CDH4 protein level in PTC cells transfected with FER1L4 vector, miR-612 inhibitor or negative control. (**D)** The relative CDH4 protein level in PTC cells co-transfected with siRNA targeting FER1L4 and miR-612 inhibitor. (**E)** The relative CDH4 protein level in PTC cells co-transfected with FER1L4 vector and miR-612 mimics. NS, not significant. Error bars, mean ± SD. * *P* < 0.05; ** *P* < 0.01; *** *P* <0.001.

**Figure S4 Quantitative analysis of the oncogenic effects of CDH4 on PTC cells.**


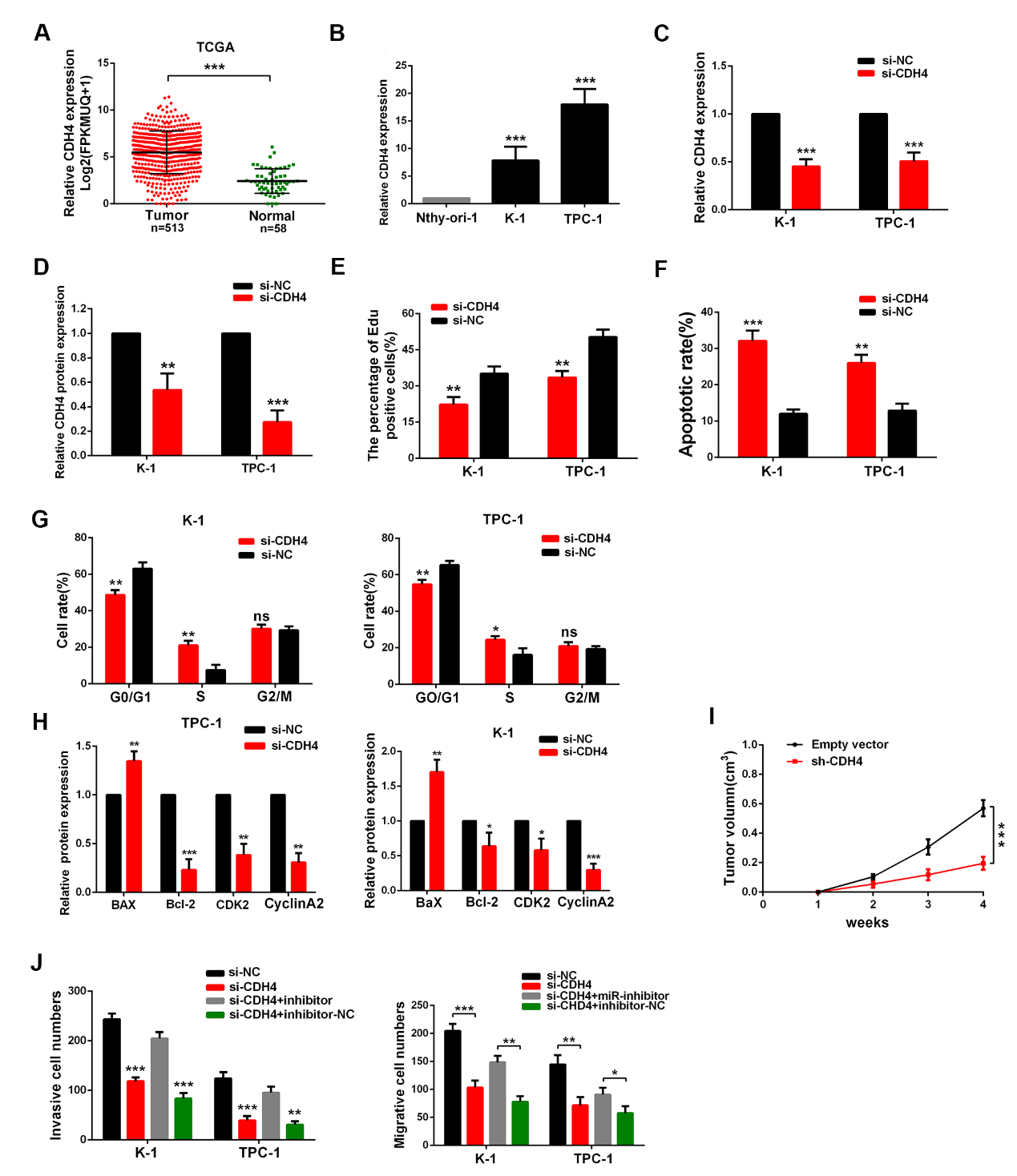


**Figure S4 Quantitative analysis of the oncogenic effects of CDH4 on PTC cells. (A)** The expression level of CDH4 in PTC tissues compared with normal tissues from TCGA database. (**B)** qRT-PCR analysis of the expression of CDH4 in K-1 and TPC-1 cells compared with Nthy-ori-1. (**C, D)** CDH4 was efficiently silenced by siRNA both at RNA level and protein level. (**E)** Cell proliferative ability was evaluated by EdU incorporation assays in PTC cells treated with siRNA targeting CDH4. (**F, G)** Flow cytometric analysis of cell apoptosis and cell cycle arrest after knockdown of CDH4. (**H)** The expression level of Bax, Bcl-2, CDK2, and CyclinA2 in CDH4-depleted TPC-1 and K-1 cells. (**I)** Relative tumor volume in implanted tumors as indicated. (**J)** miR-612 rescued the effects of CDH4 on cell invasion and migration as indicated by transwell assays. NS represented not significant. Error bars, mean ± SD. * *P* < 0.05; ** *P* < 0.01; *** *P* < 0.001.

**Figure S5** **GSEA analysis of FER1L4 and CDH4, and correlation analysis between cadherins and FER1L4.**


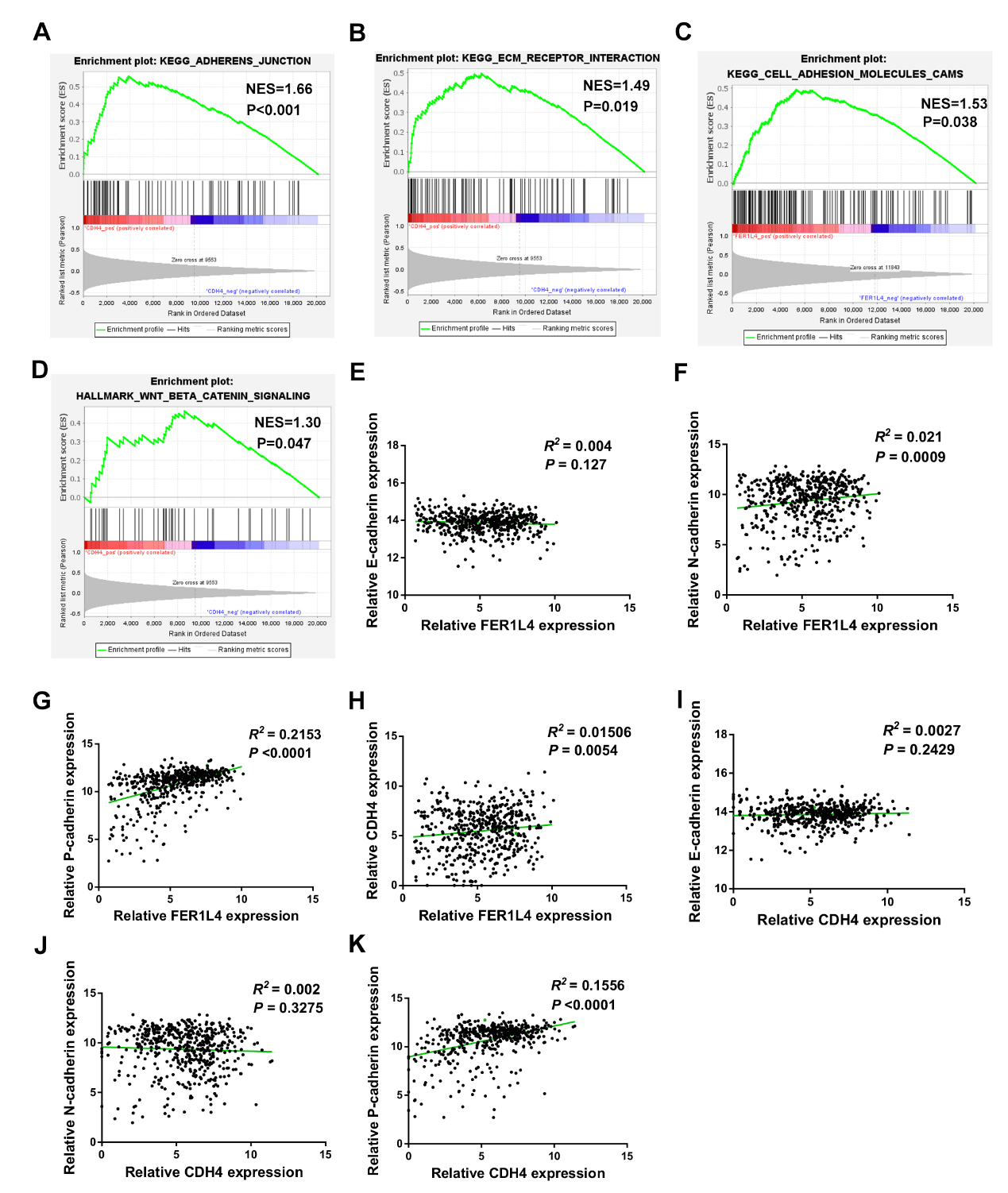


**Figure S5 GSEA analysis of FER1L4 and CDH4, and correlation analysis between cadherins and FER1L4.** According to gene expression data from the TCGA database, GSEA analysis of CDH4 and FER1L4 was conducted using gene sets database of c2.kp.kegg (**A-C**) and h.all.v.7.2 (**D**). Pearson correlation analysis of E-cadherin (**E**), N-cadherin (**F**), P-cadherin (**G**), CDH4 (**H**), and FER1L4. Pearson correlation analysis of E-cadherin (**I**), N-cadherin (**J**), P-cadherin (**K**), and CDH4.
